# Cross-sectional study of characteristics of body composition of 24,845 children and adolescents aged 3–17 years in Suzhou

**DOI:** 10.1186/s12887-023-04134-7

**Published:** 2023-07-13

**Authors:** Yan Zhao, Jin-xin Gong, Yi-ting Ji, Xiao-yun Zhao, Lu He, Shi-zhong Cai, Xiang-ming Yan

**Affiliations:** 1 Department of Pediatrics, The Second Affiliated Hospital of Soochow University, Suzhou, Jiangsu 215003 China; 2 Department of Child and Adolescent Healthcare, Children’s Hospital of Soochow University, Suzhou, 215025 Jiangsu China; 3 Department of Neonatology, Children’s Hospital of Soochow University, Suzhou, 215025 Jiangsu China; 4 Department of Urology, Children’s Hospital of Soochow University, Suzhou, 215025 Jiangsu China

**Keywords:** Body composition, Child, Fat mass, Fat-free mass

## Abstract

**Background:**

We aimed to analyze the characteristics of the body composition of children and adolescents aged 3–17 in Suzhou, China.

**Methods:**

A cross-sectional study between January 2020 and June 2022 using bioelectrical impedance was conducted to determine the fat mass (FM), fat-free mass (FFM), skeletal muscle mass, and protein and mineral contents of 24,845 children aged 3–17 who attended the Department of Child and Adolescent Healthcare, Children’s Hospital of Soochow University, China. Measurement data was presented in tables as mean ± *SD*, and groups were compared using the independent samples *t*-test.

**Results:**

FM and fat-free mass increased with age in both boys and girls. The fat-free mass of girls aged 14–15 decreased after reaching a peak, and that of boys in the same age group was higher than that of the girls (*p* < 0.05). There were no significant differences in FM between boys and girls younger than 9- and 10-years old. The percentage body fat (PBF) and FM index of girls increased rapidly between 11 and 15 years of age (*p* < 0.05), and those of boys aged 11–14 were significantly lower (*p* < 0.05), suggesting that the increase in body mass index (BMI) was mainly contributed by muscle mass (MM) in boys.

**Conclusions:**

The body composition of children and adolescents varies according to their age and sex. A misdiagnosis of obesity made on the basis of BMI alone can be avoided if BMI is used in combination with FM index, percentage body fat, and other indexes.

**Supplementary Information:**

The online version contains supplementary material available at 10.1186/s12887-023-04134-7.

## Background

In the past 40 years, the numbers of children and adolescents with obesity have increased rapidly from 11 million to 124 million, and this represents a substantial threat to public health worldwide [1]. Currently, body mass index (BMI) is widely accepted as a gold standard for the evaluation of nutritional status, and is recommended by the World Health Organization as a suitable index for screening populations for overweight and obesity, because of its convenience and efficiency [2]. However, BMI has its limitations. For example, it does not accurately reflect changes in body fat content and distribution; in particular, skeletal muscle mass (SMM) increases rapidly and body mass increases significantly in adolescent boys. Indeed, the individual contributions of muscle mass (MM) and fat mass (FM) to overall body mass cannot be differentiated using BMI alone, meaning that obesity may be over-diagnosed [3].

Currently, measures of body composition are widely used in the clinic to evaluate individual nutritional status, and FM and fat-free mass (FFM) (including water, proteins, minerals, bone, and muscle) are typically quantified. MM changes according to environmental stimuli, such as diet and protein balance. Low muscle mass is associated with a number of problems, such as muscle weakness, poor immunity, and a higher risk of infection [4]. In addition, persistent high FM can be associated with hyperlipidemia, diabetes, hypertension, hyperuricemia, and other diseases [2]. Thus, the correct assessment of changes in body composition is very important for the timely identification, diagnosis, and treatment of nutritional metabolic diseases [5, 6].

Many methods have been used to assess body composition, such as the measurement of skinfold thickness, magnetic resonance imaging, dual-energy X-ray absorptiometry (DXA), and bioelectrical impedance analysis (BIA). BIA is a non-invasive method that involves the passing of a low-frequency current through an organism, which encounters resistance according to the nature of the fluid and cellular structure through which it passes, and the impedance of this signal is high in adipose tissue and low in lean tissue. BIA proved to be an effective and reliable scale for body composition assessment [7–10]. Because of the simplicity, low price, safety, and non-invasiveness of this technique, it is widely used in the clinic. From pre-school period, school age to puberty, children and adolescents undergo significant physical changes, including bone mineral accumulation, linear growth, gains in muscle and fat, sexual development and maturation. These changes of FM and FFM vary according to age and gender, and puberty process.

Fangfang Chen performed the research among 3- to 5-year-old children in Tianjin, China [11]. Ling Bai reported the results of body composition using the BIA in Chinese children (10–18 years old), mainly focus the attention on the associations of body fat distribution and lean body mass with blood pressure in normal-weight children and adolescents [12]. Liu Zhang investigated the relationship between body compositions and bone mineral density and the effect of composition substitution among Chinese children (5-18years old) [13]. However, previous analysises of body composition of children and adolescents have seldom focused on the specific changes in body composition that occurs with age, and comprehensive large samples studies were quite deficient, making it difficult to assess the growth trend and related health risks of children. To fill this gap, we designed the present study to assess body composition by BIA in Chinese children and adolescents aged 3–17 years in Suzhou (It is located between 119 ° $55^{\prime}$ - 121 ° $20^{\prime}$ E and 30 ° $47^{\prime}$ - 32 ° $02^{\prime}$ N, in the southeast of Jiangsu Province. The city has a low and flat terrain, crisscross rivers and numerous lakes. There are four distinct seasons with mild climate and abundant rainfall), China, to characterize the changes in various age groups and provide evidence-based guidance regarding diet and exercise for children, to promote their healthy development.

## Methods

### Study participants

The participants were selected from children aged 3–17 years who underwent a physical examination between January 2020 and June 2022 from the Department of Child and Adolescent Healthcare, the Children’s Hospital of Soochow University, China. The inclusion criteria were: (1) availability of a complete set of basic demographic information (age and sex) ;(2) availability of a complete set of data from the physical examination (height, body mass, and body composition). The exclusion criteria were the presence of severe chronic disease, genetically determined endocrine or metabolic diseases, or chromosomal or genetic abnormalities. Ultimately, 24,845 participants were enrolled, they were grouped on the basis of their age, for example, if the child was 3-3.9 years old, he belonged to the 3 years old group. The study was approved by the Ethics Committee of the Children’s Hospital of Soochow University (No:2021CS092) and informed consent was obtained from all participants or, if participants were under 16, from a parent and legal guardian.

### Anthropometry

The children wore a single layer of light clothing and stood against the wall. Their height and body mass were measured twice using standard methods to 0.1 cm and 0.1 kg, respectively. BMI was calculated as body mass (kg)/height (m)^2^.

### Determination of body composition

Body composition was assessed using a body composition analyzer (Inbody J 20, Inbody, Seoul, Korea) that functions using the principle of BIA. It measures impedance by applying alternating current to human body [14]. The contact resistance will occur when the human body contacts with the electrode. InBodyJ20 frequency ranges from 5, 50 to 250 kHz, and it is phase sensitive, high frequency is more suitable for measuring intracellular fluid, low frequency is better fit for measuring extracellular fluid [15–17]. Multi-frequency is widely used in clinical for analysis of individual body composition [18]. This procedure was performed by professionally trained operators. The device was calibrated half an hour before use every day. The children emptied their bladders at this time, and the consumption of water was not permitted within the half-hour preceding the measurement. They wore a single layer of clothing without shoes or socks, and stood in the appropriate position. First, using the InBody tissue to wet the participants’ hands and feet, then standing on the InBody to measure the weight and ensure that the heel keep straight with the foot electrode. After entering the name, gender, age, and height, the measurement began. The participants were required to grasp the handle and place the thumb on the oval electrode, keep arms straight and do not touch other parts of the body. The whole process lasted about 1 min. FM, FFM, MM, skeletal muscle mass (SMM), percentage body fat (PBF), waist-to-hip ratio (WHR) were recorded; and the fat mass index (FMI), fat-free mass index (FFMI), and skeletal muscle mass index (SMMI) were calculated by dividing the FM, FFM, and SMM by the square of height (m^2^).

### Statistics

Data were entered and verified by two people. SPSS v.27.0 (IBM, Inc., Armonk, NY, USA) was used to analyze the data. Measurement data was presented in tables as mean ± *SD* in Supplementary Tables 1, 2 and 3, and 95, 90, 75, 50, 25, 10, and 5 percentile cut-off values were shown in Supplementary Table 4, differences among genders were compared by independent samples *t*-test or Mann-Whitey *U* test. Kruskal-wallis was used to compare multiple groups, followed by the least significant difference test. Two-sided *p* < 0.05 was considered to represent statistical significance.

## Results

### Comparison of indices of body composition in boys and girls

A total of 24,845 children and adolescents aged 3–17 years were studied (8,830 girls and 16,015 boys). The height, body mass, protein content, mineral content, bone mineral content, FM, MM, FFM, SMM, and BMI of the boys were significantly higher than those of the girls, but the PBF of the boys was significantly lower (*p* < 0.05), as shown in Table 1.

**Table 1:** Basic anthropometric and BIA data for the study group

	Girls (*n* = 8,830)	Boys (*n* = 16,015)	t	*P*
Age(years)	8.05 ± 2.49	8.40 ± 2.71	132.14	0.00
Height (cm)	127.04 ± 15.68	129.90 ± 17.30	166.64	0.00
Weight (kg)	29.26 ± 11.93	32.08 ± 14.58	240.31	0.00
BMI (kg/m^2^)	17.44 ± 3.54	18.12 ± 4.01	178.81	0.00
Protein (kg)	4.22 ± 1.34	4.70 ± 1.79	500.68	0.00
Minerals (kg)	1.53 ± 0.55	1.68 ± 0.71	301.12	0.00
Bone mineral content (kg)	1.27 ± 0.47	1.39 ± 0.61	265.68	0.00
FFM (kg)	21.63 ± 6.89	24.06 ± 9.09	479.2	0.00
SMM (kg)	10.73 ± 4.05	12.20 ± 5.38	499.957	0.00
MM (kg)	20.36 ± 6.46	22.67 ± 8.54	489.83	0.00
FM (kg)	7.59 ± 5.82	8.04 ± 6.82	26.66	0.00
PBF (%)	23.68 ± 8.97	22.52 ± 9.53	87.94	0.00

*Abbreviations:* *BMI* Body mass index, *FFM* Fat free mass, *SMM* Skeletal muscle mass, *MM* Muscle mass, *FM* Fat mass, *PBF* Percentage body fat, *t*-test was used to compare the data

### Comparison of the major body components of boys and girls of the same age

**Fig. 1:**
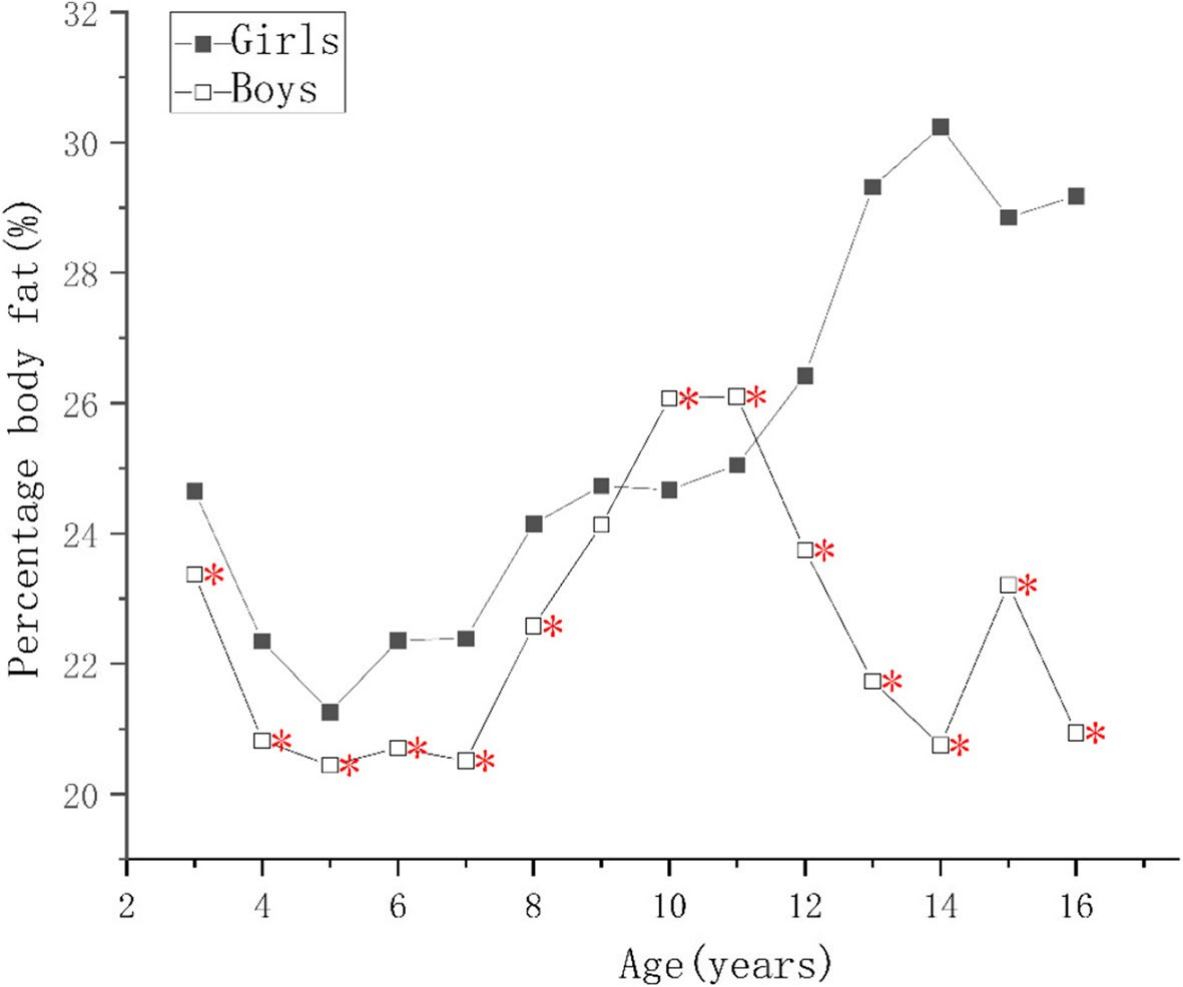
Figure Changes in indexes of body composition in children aged 3–17 years in Suzhou, China. Comparison of the main body composition indexes among boys and girls at various ages, * indicate the differences are statistical significant, where *: *p*< 0.05

**Fig. 2:**
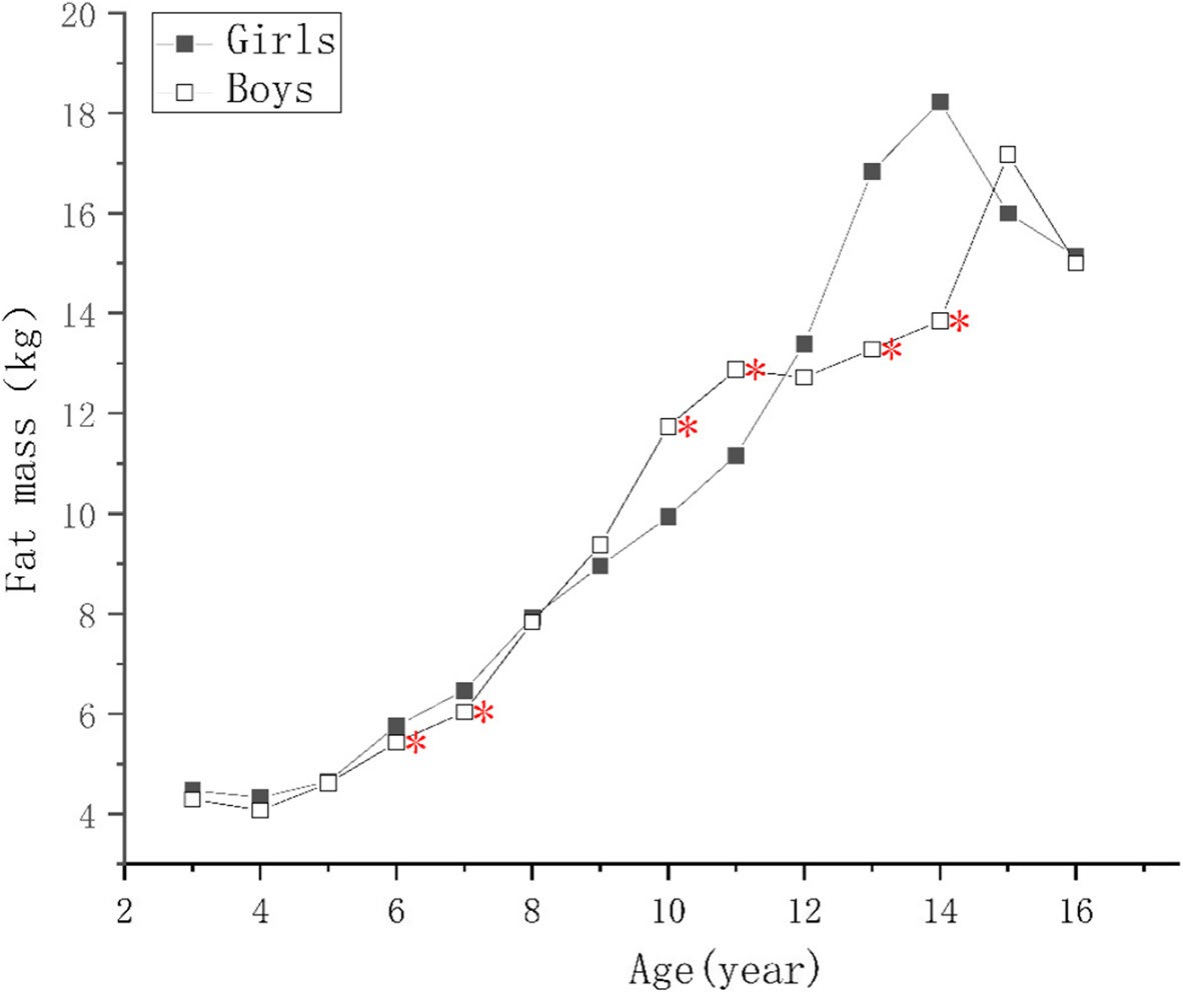
Figure Changes in indexes of body composition in children aged 3–17 years in Suzhou, China. Comparison of the main body composition indexes among boys and girls at various ages, * indicate the differences are statistical significant, where *: *p* < 0.05

**Fig. 3:**
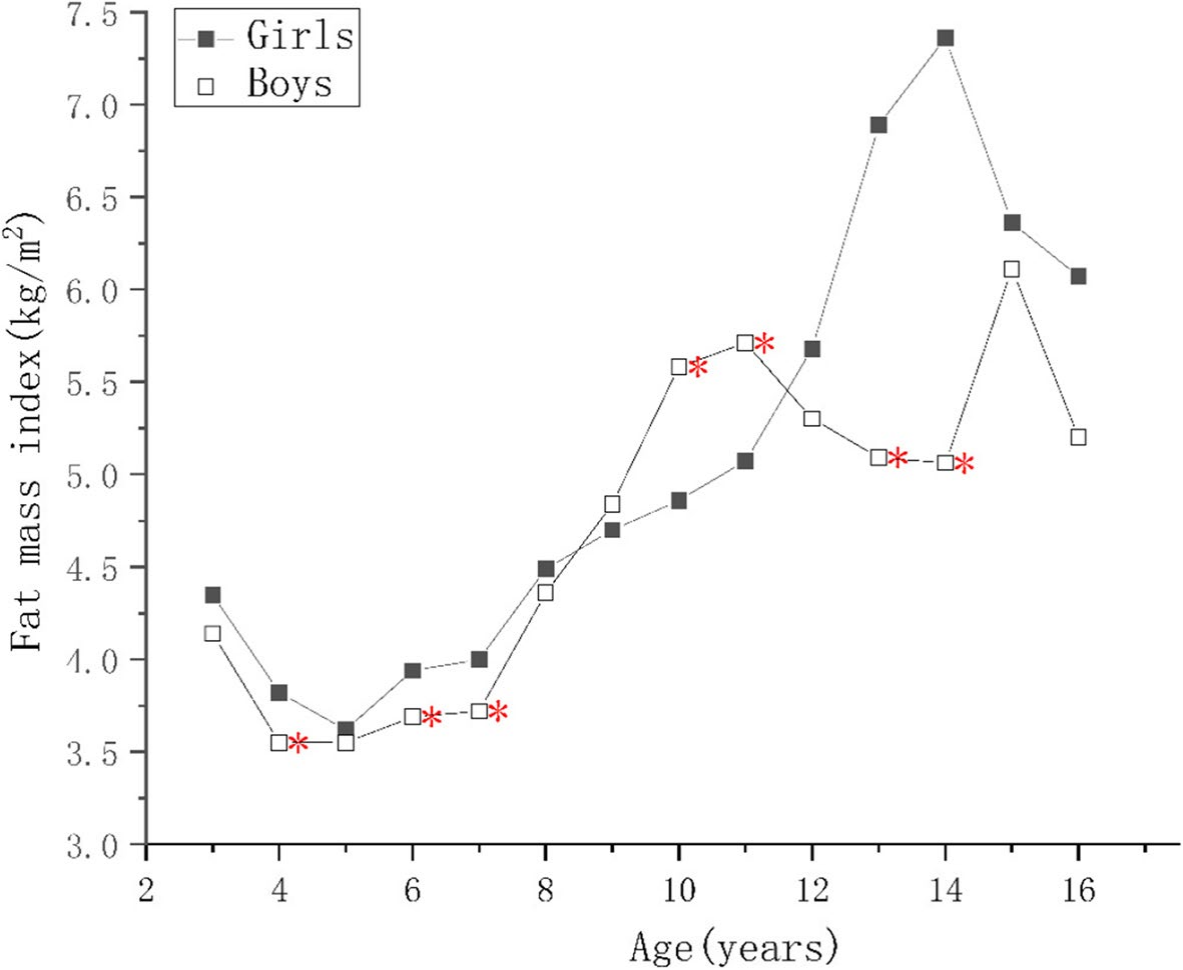
Figure Changes in indexes of body composition in children aged 3–17 years in Suzhou, China. Comparison of the main body composition indexes among boys and girls at various ages, * indicate the differences are statistical significant, where *: *p* < 0.05

For children of the same ages, the protein, mineral, and bone mineral contents; and the MM, SMM, SMMI, FFM, FFMI of the boys were higher than those of the girls (*p* < 0.05) as shown in Supplementary Tables 1, 2 and 4. There were no significant differences in the PBF of the boys and girls aged 9–10 years (*p* > 0.05), but it was higher in girls of other ages (*p* < 0.05), as shown in Supplementary Tables 3 and Fig. 1. The FM of girls aged 6–8 and 13–15 years were higher than those of boys (*p* < 0.05), boys’ FM were higher than girls in 11–12 and 13–14 age groups (*p* < 0.05), but there were no differences in the other age groups (*p* > 0.05), as shown in Supplementary Table 2 and Fig. 2. The FMI of girls aged 4–5, 6–8, and 13–15 years were higher than those of boys and the FMI of boys of 10–12 years of age was higher than that of the girls (*p* < 0.05), but there were no significant differences in the other age groups (*p* > 0.05), as shown in Fig. 3.

### Trends in body composition with age

**Fig. 4:**
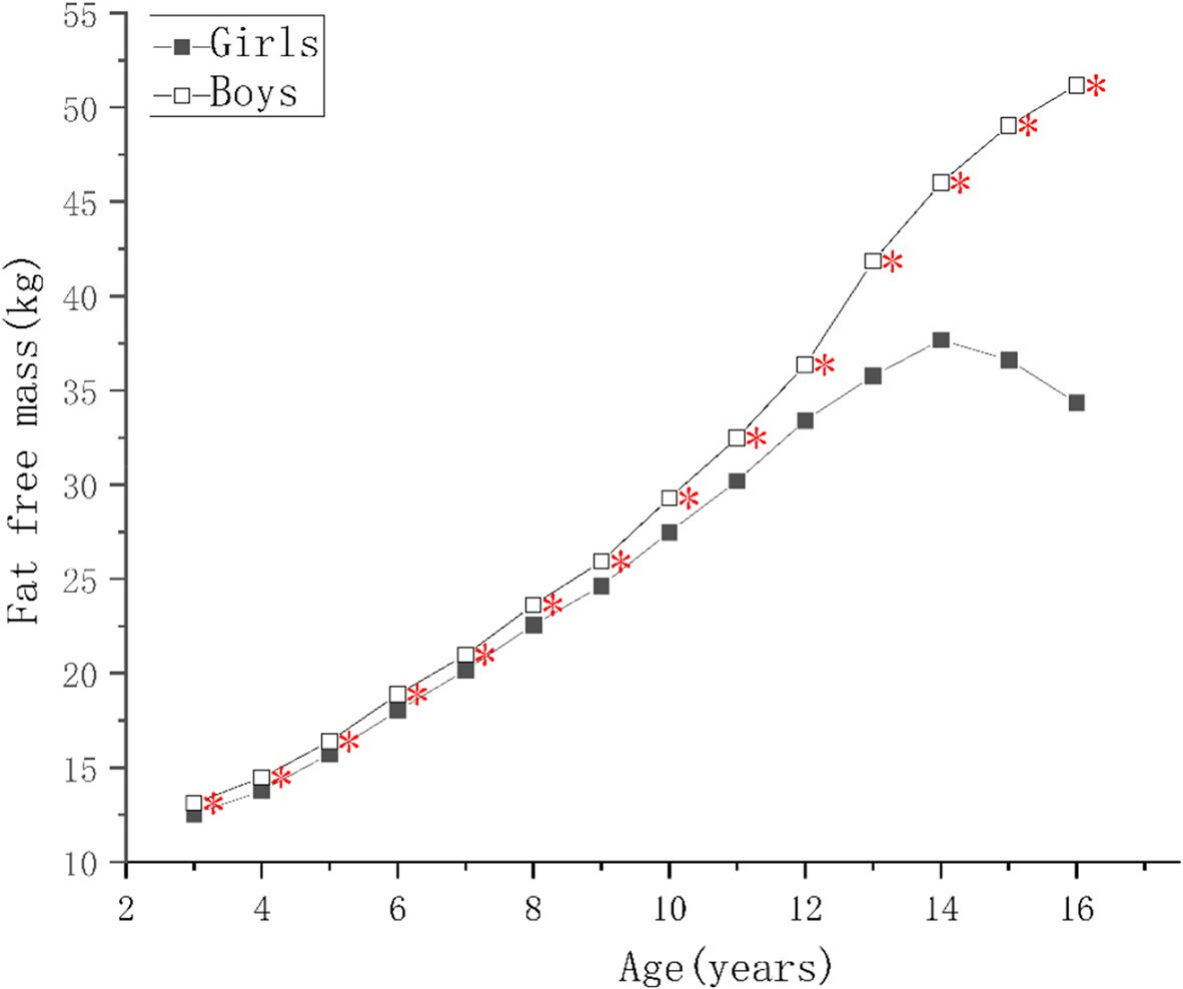
Figure Changes in indexes of body composition in children aged 3–17 years in Suzhou, China. Comparison of the main body composition indexes among boys and girls at various ages, * indicate the differences are statistical significant, where *: *p* < 0.05

**Fig. 5:**
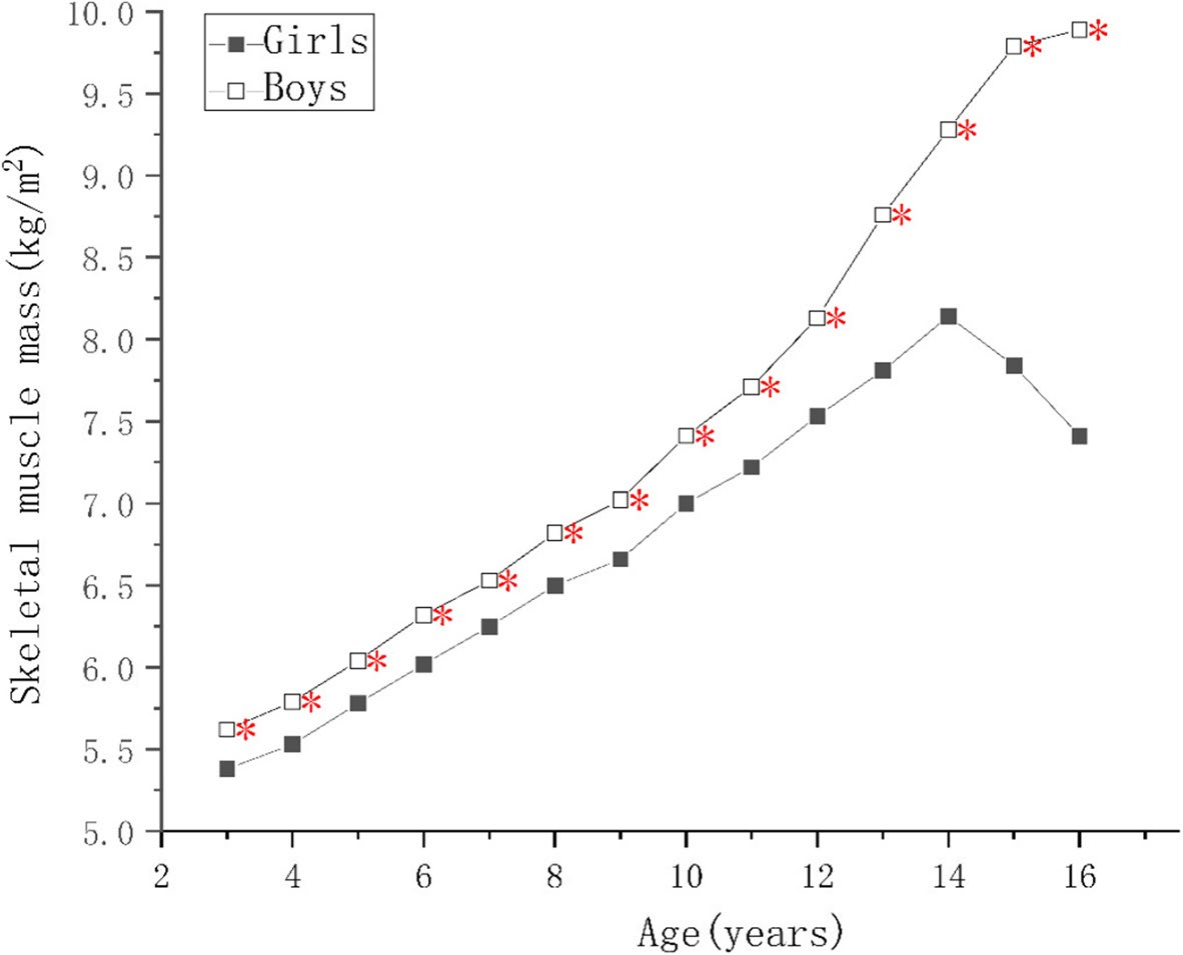
Figure Changes in indexes of body composition in children aged 3–17 years in Suzhou, China. Comparison of the main body composition indexes among boys and girls at various ages, * indicate the differences are statistical significant, where *: *p* < 0.05

As the children got older, the protein, mineral, and bone mineral contents; and the MM, SMM, SMMI, and FFM of girls increased year by year (*p* < 0.05), reaching a peak at 14–15 years of age, and slowly decreasing thereafter, as shown in Fig. 4. There was no significant increase in FFMI in girls younger than 5 years of age, and the change was similar to that of FFM in children of > 5 years of age. The protein content, MM, FFMI, SMM, and SMMI of the boys continued to increase as they got older, and the rates of the changes were greater than those of the girls (*p* < 0.05). Each index tend to grow slower after the age of 15, and the difference was not statistically significant (*p* > 0.05), as shown Figs. 4 and 5. The FM and FMI of both boys and girls aged 3–5 years were stable, but PBF decreased (*p* < 0.05). After the age of 7 years, the FM of both the boys and girls increased rapidly, but in children younger than 10–12 years of age, the mean value in boys was higher than in girls. However, after the age of 12 years, the FM of the girls increased faster and surpassed boys (*p* < 0.05). The FM of the girls and boys decreased after reaching peaks at 14 and 15 years of age, respectively. The PBF and FMI of the boys and girls showed similar changes below the age of 11, but those of girls increased rapidly at the ages of 5–6, 7–9, and 11–15 (*p* < 0.05), but did not change at the ages of 6–7 or 9–10. Their values peaked at the ages of 14 and 15, respectively, and then gradually decreased. The PBF and FMI of boys did not significantly change at the ages of 6–7 and 10–11 years, but rapidly increased (*p* < 0.05) at the ages of 7–10 and 14–15 years, and rapid decreased (*p* < 0.05) at the age of 11–14 years old, more substantially with respect to PBF. A comparison of these body composition indexes, according to sex and age, is shown in Supplementary Tables 1, 2, 3 and 4.

## Discussion

Body composition analysis is an important method of evaluating the influences of diet, exercise, diseases, growth, and development on the human body. The components of the human body that can be quantified include water, protein, minerals, and fat. Specifically, FM can be used to predict the risk of obesity, hypertension, type 2 diabetes, cardiovascular disease, and other diseases; and the FFM is important for exercise tolerance and balance [19–21]. A good understanding of appropriate body composition and changes is important for health education, dietary guidance, and physical exercise recommendations during childhood development.

The body composition and distribution of materials and tissues differ significantly as age, gender difference, and puberty development varies accordingly [22]. During most of their childhood, the growth rates of boys and girls are similar. The mean height, body mass, FM, and FFM of boys are slightly higher than those of girls, whereas the FMI of boys is lower than that of girls. From childhood to adolescence, boys and girls gradually manifest huge differences in physical, psychological, and behavioral parameters because of differing secretion of sex hormones. However, the values of indexes of body composition, such as FM and FFM, change differently.

Luteinizing hormone (LH) and follicle-stimulating hormone (FSH) in girls stimulate uterine growth and the secretion of estrogen and small amounts of androgens. Under the influence of sex hormones, most girls start to appear secondary sexual characteristics at the age of 9–10, such as breast enlargement, pubic and underarm hair growth, internal and external genital development, and menarche [23]. In addition, high blood estrogen concentrations have direct effects on epiphyses and indirectly stimulate an increase in pulsatile secretion of growth hormone (GH) by the pituitary gland, which causes increases in bone mineral content and skeletal muscle mass [24]. However, estrogen has a bidirectional effect on bone growth: at a low concentration, it accelerates osteogenesis in the cartilage through activation of the growth hormone/insulin-like growth factor-1 axis, which increases the linear growth [25]; whereas when present at a high concentration during late puberty, it binds to receptors in growth plates, promoting epiphyseal closure [26]. Another characteristic change during adolescence is a rapid increase in FM, mainly in the chest, abdomen, and hips, which makes the bodies of girls rounder, and their body composition gradually approach those of adults [25].

Most boys enter adolescence at 11–12 years old, when LH and FSH stimulate the testes to secrete androgens and a small amount of estrogen, which results in a series of changes including testicular enlargement, penis development, the growth of pubic and underarm hair, voice change, and spermatorrhea. Unlike girls, androgens cause a rapid increase in SMM from early puberty in boys, and their proportion of fat decreases, so that the “triangular” male body shape, featuring stronger shoulders and chest, gradually develops. In contrast, PBF shows a downward trend. In late puberty, height growth decelerates, all body functions are well developed [27].

In the present study, the body composition of 24,845 children and adolescents of 3–17 years of age was characterized cross-sectionally in Suzhou, China. The FM and FFM of boys and girls of different ages vary significantly. In addition, we found that the protein, mineral, and bone mineral content; and the MM, SMM, SMMI, and FFM of boys were higher than those of girls, and steadily increased with age. These indexes peaked at 14–15 years old in girls, after which they decreased, and the girls reached physical maturity earlier than the boys. After the age of 15, the protein content and skeletal muscle mass of girls decreased year by year. Therefore, it is necessary to appropriately reduce their daily calorie intake and they should avoid the excessive intake of high-fat and high-protein food. However, boys of the same age should continue to consume an appropriate diet to maximize their growth potential.

**Fig. 6:**
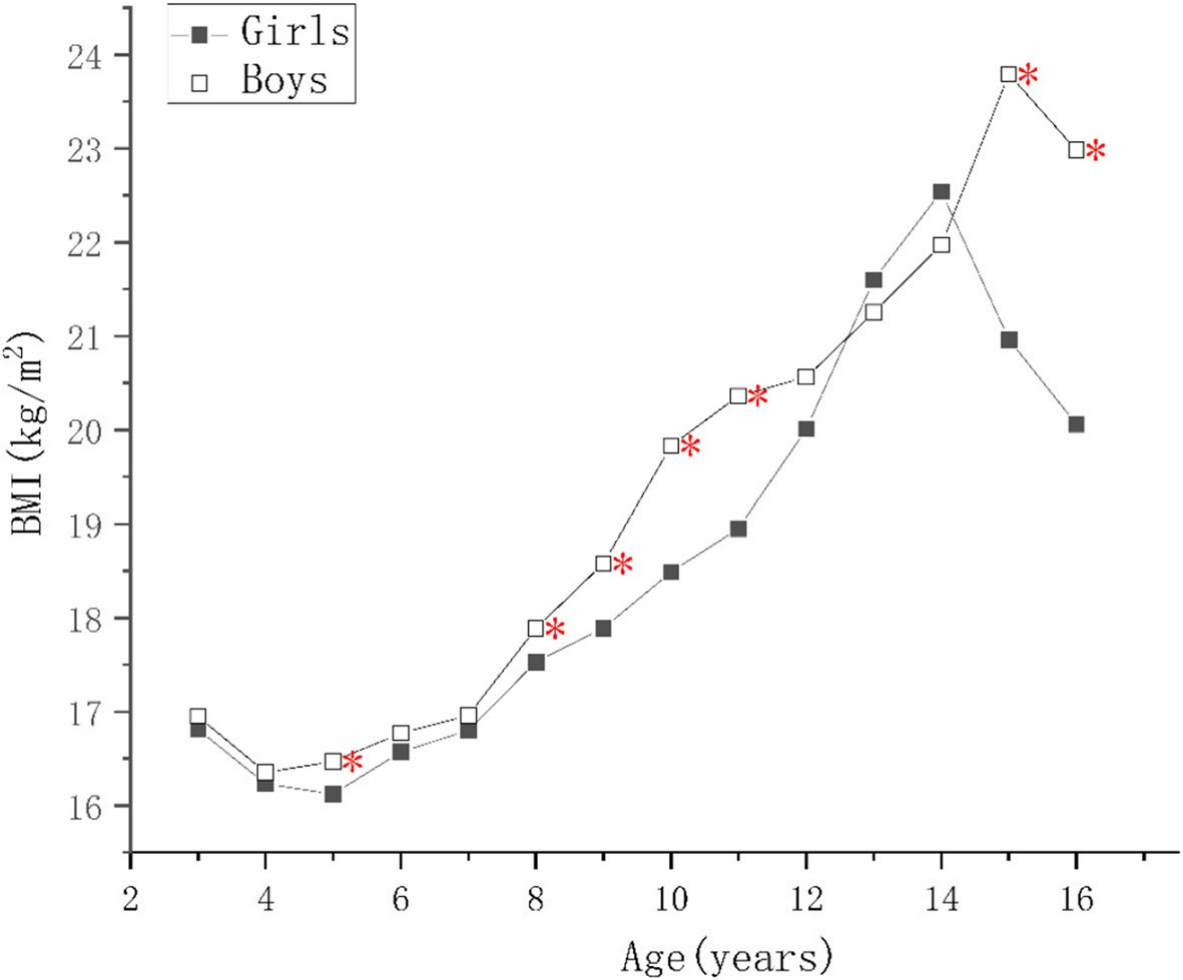
Figure Changes in indexes of body composition in children aged 3–17 years in Suzhou, China. Comparison of the main body composition indexes among boys and girls at various ages, * indicate the differences are statistical significant, where *: *p* < 0.05

Boys and girls have similar FM before the age of 9 years, and although these grow faster in boys than in girls at the age of 9–11 years, it increases faster in girls after the age of 12. There are also significant differences in the PBF and FMI of boys and girls. In the present study, we have shown that the values of these indexes in boys and girls significantly decrease at the age of 3–5 years, which is consistent with the results of a previous analysis of the body composition of 3,593 healthy individuals aged 4–24 years in Germany [28]. Besides, data from 1011 children aged 3–5 years old in Tianjin [11], And research of 1243 preschool children in Xiamen, China [29], also showed declining trend of FMI, and FM% among both boys and girls. Thus, it appears that there are similarities in the changes of body fat percentage in different regions, countries and ethnicities. These changes may be explained by the significant increase in physical activity and the accompanying increase in skeletal muscle mass over 3 years old. In the present study, PBF in girls were higher than that in boys in every age group, except for the 9- and 10-years old groups (all *P* < 0.05), which was in line with F F Chen’s results conducted in seven cities of China [30]. PBF and FMI increase rapidly again in girls aged 7–9 years, which is closely associated with the onset of puberty. In addition, at the age of 11–15 years, FM continues to increase rapidly, peaking at approximately 15 years of age, after which it gradually decreases, which suggests that FM increases more substantially during mid- and late puberty. PBF and FMI decrease rapidly in boys aged 11–14 years, whereas FM continues to increase, which may be because boys have entered puberty by this age. During this period, androgens cause a rapid increase in muscle mass which exceeds the fat mass, resulting in a decline in the proportion of fat. Furthermore, the BMI of boys shows a rapid upward trend after the age of 11 years, as shown in Fig. 6. BMI is known as an indicator of adiposity; however, both FMI and FFMI affect BMI, making it impossible to differentiate body fat from lean mass using BMI alone [31, 32]. Manfred James Müller et al. [33] demonstrated both FM and FFM increase with a rising BMI, and correlation coefficients relating BMI to FM and FFM were similar, but regression lines differ in slopes. In addition, the association between BMI and PBF was curve-linear. Hye Won Park et, al. [34] reported decreases in FMI and PBF despite increases in BMI, and a large increase in FFMI was observed in males in Korean. Demerath, et al. [35] explained that boys experience greater gains in muscle and lean mass than in fat mass. Higher BMI scores indicate that an individual has relatively more weight-for-height than a person with a lower score. The value of BMI indicates only this and does not provide any information about FM or FFM. So, BMI is actually less than ideal for measuring obesity [36, 37]. The BMI of girls and boys showed an upward trend before the age of 14 and 15 respectively, as well as a steady growth in SMI. There were significant fluctuations in the FMI and PBF, especially during the period of 11–14 years old, when the above indicators rapidly descended among boys. Nevertheless, the BMI of boys in this age group was still on the rise, suggesting that the increase in BMI was mainly contributed by SMMI in boys. Therefore, clinicians should comprehensively analyze the body composition of boys of this age to avoid misdiagnosing them as having overweight or obesity, because of rapid increases in body mass and BMI. The changes in body composition of boys aged 9–17 years were previously assessed using DXA in a study conducted in Colombia, where it was shown that FMI continues to decrease at this age. F F Chen [30] also reported FMI decreased obviously between 10-15 years old in boys. Unlike the age group and change characteristics from the above researches, in the present study, the FMI of boys continues to decline at the age of 11–14, subsequently increased transiently at 14–15 years old and then decreased rapidly. The reasons for this discrepancy may be due to the different research methods, environments, ethnicities, or ages of the onset of puberty. In addition, the nature of the education system in China means that this increase in the FMI of adolescents at this age is associated with great pressure, overload homework, long learning sessions, and insufficient daily exercise, which may explain the increase in fat accumulation. Thus, it is necessary to improve the health education of adolescents, and to encourage a healthy lifestyle, comprising an appropriate diet, moderate exercise, and balance between activity and rest, in order to reduce the incidence of overweight and obesity during adolescence.

The strengths of the present study include its large sample size and the comprehensive analysis of the changes in body composition, according to sex and age, which should help understanding of the growth and development of children. However, the study also had some limitations: the number of participants of > 15 years of age was small, which may have caused some bias, and the Tanner stages of the participants was not used to compare their body compositions with their developmental stages.

## Conclusions

The present findings confirm that the body composition of children and adolescents changes with age, especially during puberty; and that girls show a significant increase in fat mass, whereas boys show a significant increase in muscle mass. Therefore, we suggest that the assessment of the health status of children should involve an analysis of body composition, to avoid the use of BMI alone, which can lead to a misdiagnosis of overweight or obesity.

## Supplementary Information

**Additional file 1: Supplementary Table 1.** Physical parameters and body composition of the boys and girls at various ages.

**Additional file 2: Supplementary Table 2.** Physical parameters and body composition of the boys and girls at various ages.

**Additional file 3: Supplementary Table 3.** Physical parameters and body composition of the boys and girls at various ages.

**Additional file 4: Supplementary Table 4.** Percentile cut-off values of physical parameters and body composition of  boys and girls at various ages.

## Data Availability

The datasets used and/or analyzed during the current study are available from the corresponding author on reasonable request.

## Abbreviations

BIA: Bioelectrical impedance analysis
BMI: Body mass index
FFM: Fat-free mass
FFMI: Fat-free mass index
FM: Fat mass
FMI: Fat mass index
MM: Muscle mass
PBF: Percentage body fat
SMM: Skeletal muscle mass
WHR: Waist-to-hip ratio

## References

[1] NCD Risk Factor Collaboration (2017). Worldwide trends in body-mass index, underweight, overweight, and obesity from 1975 to 2016: a pooled analysis of 2416 population-based measurement studies in 128.9 million children, adolescents, and adults. Lancet.

[2] WHO Expert Consultation (2004). Appropriate body-mass index for Asian populations and its implications for policy and intervention strategies. Lancet.

[3] Mastorci F, Vassalle C, Chatzianagnostou K, Marabotti C, Siddiqui K, Eba AO (2017). Undernutrition and overnutrition burden for diseases in developing countries: the role of oxidative stress biomarkers to assess disease risk and interventional strategies. Antioxodants.

[4] Gamrin-Gripenberg L, Sundström-Rehal M, Olsson D, Grip J, Wernerman J, Rooyackers O (2018). An attenuated rate of leg muscle protein depletion and leg free amino acid efflux over time is seen in ICU long-stayers. Crit Care.

[5] Holmes CJ, Racette SB (2021). The Utility of Body Composition Assessment in Nutrition and Clinical Practice: an overview of current methodology. Nutrients.

[6] Borga M, West J, Bell JD, Harvey NC, Romu T, Heymsfield SB. Advanced body composition assessment: from body mass index to body composition profiling. J Investig Med. 2018;66(5):1–9. 10.1136/jim-2018-000722. 10.1136/jim-2018-000722 PMC5992366 29581385

[7] Larsen MN, Fristrup P, Araújo Póvoas SC, Castagna C (2021). Accuracy and reliability of the InBody 270 multi-frequency body composition analyser in 10–12-year-old children. PLoS One.

[8] Lim JS, Hwang JS, Lee JA, Kim DH, Park KD, Jeong JS (2009). Cross-calibration of multi-frequency bioelectrical impedance analysis with eight-point tactile electrodes and dual-energy x-ray absorptiometry for assessment of body composition in healthy children aged 6–18 years. Pediatr Int.

[9] Delshad M, Beck KL, Conlon CA, Mugridge O, Kruger MC, von Hurst PR (2021). Validity of quantitative ultrasound and bioelectrical impedance analysis for measuring bone density and body composition in children. Eur J Clin Nutr.

[10] Wang L, Hui SS (2015). Validity of Four Commercial Bioelectrical Impedance Scales in Measuring Body Fat among Chinese Children and Adolescents. Biomed Res Int.

[11] Chen F, Wang J, Liu J, Huang G, Hou D, Liao Z (2022). Characteristics of body composition estimated by Air-Displacement Plethysmography in Chinese Preschool Children. Front Public Health.

[12] Bai L, Zhou J, Tong L, Ding W (2022). Association between body composition and blood pressure in normal-weight Chinese children and adolescents. BMC Pediatr.

[13] Zhang L, Li H, Zhang Y, Kong Z, Zhang T, Zhang Z (2021). Association of Body Compositions and Bone Mineral Density in Chinese Children and Adolescents: Compositional Data Analysis. Biomed Res Int.

[14] Harder R, Diedrich A, Whitfield JS, Buchowski MS, Pietsch JB, Baudenbacher FJ (2016). Smart multi-frequency bioelectrical impedance spectrometer for BIA and BIVA applications. IEEE Trans Biomed Circuits Syst.

[15] Simpson JA, Lobo DN, Anderson JA, Macdonald IA, Perkins AC, Neal KR (2001). Body water compartment measurements: a comparison of bioelectrical impedance analysis with tritium and sodium bromide dilution techniques. Clin Nutr.

[16] Jaffrin MY, Morel H (2008). Body Fluid volumes measurements by impedance: a review of bioimpedance spectroscopy (BIS) and bioimpedance analysis (BIA) methods. Med Eng Phys.

[17] Nescolarde L, Yanguas J, Lukaski H, Rodas G, Rosell-Ferrer J (2014). Localized BIA identifies structural and pathophysiological changes in soft tissue after post-traumatic injuries in soccer players. Annu Int Conf IEEE Eng Med Biol Soc.

[18] Marra M, Sammarco R, De Filippo E, De Caprio C, Speranza E, Contador F (2019). Resting Energy Expenditure, Body Composition and Phase Angle in Anorectic, Ballet Dancers and constitutionally lean males. Nutrients.

[19] Zhang L, Chen R, Li R, Chen M-Y, Huang R, Li X-N (2018). Evaluating the predictive factors of resting energy expenditure and validating predictive equations for Chinese obese children. World J Pediatr.

[20] van Beijsterveldt IALP, van der Steen M, de Fluiter KS, Spaans SAMJ, Hokken-Koelega ACS (2022). Body composition and bone mineral density by Dual Energy X-ray absorptiometry: reference values for young children. Clin Nutr.

[21] Ji Y-T, Li L-L, Cai S-Z, Shi X-Y (2022). Body composition in preschool children with short stature: a case-control study. BMC Pediatr.

[22] Pezoa-Fuentes P, Cossio-Bolaños M, Urra-Albornoz C, Alvear-Vasquez F, Lazari E, Urzua-Alul L (2023). Fat-free mass and maturity status are determinants of physical fitness perform. J Pediatr (Rio J).

[23] Haapala EA, Haapala HL, Syväoja H, Tammelin TH, Finni T, Kiuru N (2020). Longitudinal associations of physical activity and pubertal development with academic achievement in adolescents. J Sport Health Sci.

[24] Binder G, Iliev DI, Dufke A, Wabitsch M, Schweizer R, Ranke MB (2005). Dominant transmission of prepubertal gynecomastia due to serum estrone excess: hormonal, biochemical, and genetic analysis in large kindred. J Clin Endocrinol Metab.

[25] Wood CL, Lane LC, Cheetham T (2019). Puberty: normal physiology (brief overview). Best Pract Res Clin Endocrinol Metab.

[26] Nilsson O, Chrysis D, Pajulo O, Boman A, Holst M, Rubinstein J (2003). Localization of estrogen receptors-alpha and -beta and androgen receptor in the human growth plate at different pubertal stages. Endocrinol.

[27] Imboden MT, Welch WA, Swartz AM, Montoya AHK, Finch HW, Harber MP (2017). Reference standards for body fat measures using GE dual energy x-ray absorptiometry in caucasian adults. PLoS On.

[28] Schmidt SC, Bosy-Westphal A, Niessner C, Woll A (2019). Representative body composition percentiles from bioelectrical impedance analyses among children and adolescents. The MoMo study. Clin Nutr.

[29] Xu LJ, Zeng GZ, Yang MF. Body composition analysis among preschool children in Xiamen City, maternal and child health care of China. Matern Child Health Care China. 2017;32(5):985–7.

[30] Chen FF, Liu JT, Huang GM, Mi J (2020). Developmental characteristics on body composition in Chinese urban children and adolescents aged 3–17 years old. Zhonghua Liu Xing Bing Xue Za Zhi.

[31] Okorodudu DO, Jumean MF, Montori VM, Romero-Corral A, Somers VK, Erwin PJ (2010). Diagnostic performance of body mass index to identify obesity as defined by body adiposity: a systematic review and meta-analysis. Int J Obes (Lond).

[32] Hattori K, Tatsumi N, Tanaka S. Assessment of body composition by using a new chart method. Am J Hum Biol. 1997;9(5):573–8. 10.1002/(SICI)1520-6300(1997)9:5%3c573::AID-AJHB5%3e3.0.CO;2-V. 10.1002/(SICI)1520-6300(1997)9:5<573::AID-AJHB5>3.0.CO;2-V 28561425

[33] Müller MJ, Braun W, Enderle J, Anja Bosy-Westphal (2016). Beyond BMI: conceptual issues related to overweight and obese patients. Obes Facts.

[34] Park HW, Yoo HY, Kim CH, Kim H, Kwak BO, Kim KS (2015). Reference values of body composition indices: the Korean National Health and Nutrition examination surveys. Yonsei Med J.

[35] Demerath EW, Schubert CM, Maynard LM, Sun SS, Chumlea WC, Pickoff A (2006). Do changes in body mass index percentile reflect changes in body composition in children? Data from the Fels Longitudinal Study. Pediatrics.

[36] Barry B, Varela-Silva MI (2012). The body mass index: the good, the bad and the Horrid. Bulletin de la Société Suisse d’Anthropologie.

[37] Bogin B. Patterns of human growth, vol. 87. 3rd ed. https://www.cambridge.org/9781108434485:87.

